# Total Hip Arthroplasty With Impacted Bone Graft on Acetabulum for Osteoporotic Acetabular Fractures: A Report of 3 Cases

**DOI:** 10.1016/j.artd.2022.10.004

**Published:** 2022-11-28

**Authors:** Yutaro Kuwahara, Ken-ichi Yamauchi, So Mitsuya, Shinsuke Takeda

**Affiliations:** Department of Orthopedic Surgery, Toyohashi Municipal Hospital, Toyohashi, Aichi, Japan

**Keywords:** Osteoporotic acetabular fracture, Primary total hip arthroplasty, Impacted bone graft, Osteoporosis

## Abstract

Osteoporotic acetabular fractures in elderly patients have recently been increasing, but the best treatment remains uncertain due to the difficulty in stabilizing these fractures with osteosynthesis. We performed total hip arthroplasty with an impacted bone graft on the acetabula of 3 elderly patients with comminuted acetabular fractures after confirming acetabular callus formation via radiographic imaging 2 months following the patients’ initial injuries. Two of the patients presented in the subacute phase after conservative treatment, and 1 patient had no history of trauma or quadrilateral surface destruction. Two patients achieved good functional results at the 3-year follow-up. Furthermore, no loosening of the prosthesis components or subsidence of the acetabular cemented cup was evident on radiographic imaging in any of the patients.

## Introduction

Comminuted acetabular fractures usually result from high-energy trauma in young patients and are relatively rare in elderly patients. However, the proportion of aging people in society has grown due to developments in medical science. The number of different osteoporotic fractures has been increasing in turn, as has the number of complex acetabular fractures resulting from trivial trauma. An epidemiological study [1] carried out over a 27-year period showed that the occurrence of acetabular fractures in elderly patients older than 60 years increased 2.4-fold between the first and second half of the study, and fractures with a displaced anterior column were more common in the elderly than in younger patients.

For younger patients, open reduction and internal fixation (ORIF) is a widely accepted treatment that provides favorable long-term results [2]. However, it is sometimes difficult to achieve sufficient reduction and stable fixation with ORIF in osteoporotic bone, and specific fracture patterns, such as medial displacement of the quadrilateral surface, dome impaction, and femoral head injury, have been reported as predictors of failure [3]. Due to such unfavorable ORIF results, several authors have advocated for primary total hip arthroplasty (THA) [[4], [5], [6]]. THA for osteoporotic acetabular fractures requires supplemental devices and/or ORIF because less bone stock is available to support the acetabular cup, which is prone to instability due to the fracture. The procedure requires a long operative time and is known to be associated with increased intraoperative bleeding, which poses a high risk of postoperative complications. The best treatment for acetabular fractures in the elderly is therefore uncertain.

The acetabular impacted bone graft (IBG) technique is generally employed for bone defects in revision hip arthroplasties with the aim of reconstituting the bone stock [7,8]. We present the clinical and radiographic results of 3 cases treated with THA and IBG for comminuted osteoporotic acetabular fractures. We applied the IBG technique to compensate for the lack of bone stock in acetabular cups in these fragile comminuted acetabular fractures.

## Case histories

We obtained written informed consent to publish the case report from all the patients or their families, as appropriate.

All the operations were performed by 1 surgeon who had at least 10 years of hip surgery experience (K.Y.). All the patients underwent surgery under general anesthesia and were placed in the lateral position for a posterolateral approach. After resecting the femoral neck and incising a part of the anterior superior capsule, we assessed the acetabular roof's instability by manually pressing the acetabulum. The acetabular fractures in all the patients were confirmed to be stable. Granulation tissue and cartilage were resected as much as possible with bone rongeurs, and spherical reaming was performed only to remove the residual cartilage.

Approximately 5-10 mm of morselized bone chips made from the resected femoral neck with a hand rongeur were tightly compacted into the contained acetabular cavity with hemispherical impactors and a metal hammer. Depending on the size of the acetabular cavity, we added femoral head allografts. We used no allografts in case 1, 1 in case 2, and 2 in case 3.

The femoral head allografts were obtained from the patients at the time of the primary THA for osteoarthritis. Prior to collection, we checked to confirm that the patients had no blood-borne infections such as hepatitis B or C, human immunodeficiency disease, or human T-cell leukemia virus-1. The tissues were quarantined at −80°C for at least 3 months. The deep-frozen allografts were sterilized by pasteurization using a Lobator SD-2 (Telos, Marburg, Germany) and then ground into morselized bone chips with a hand rongeur.

**Figure 1:**
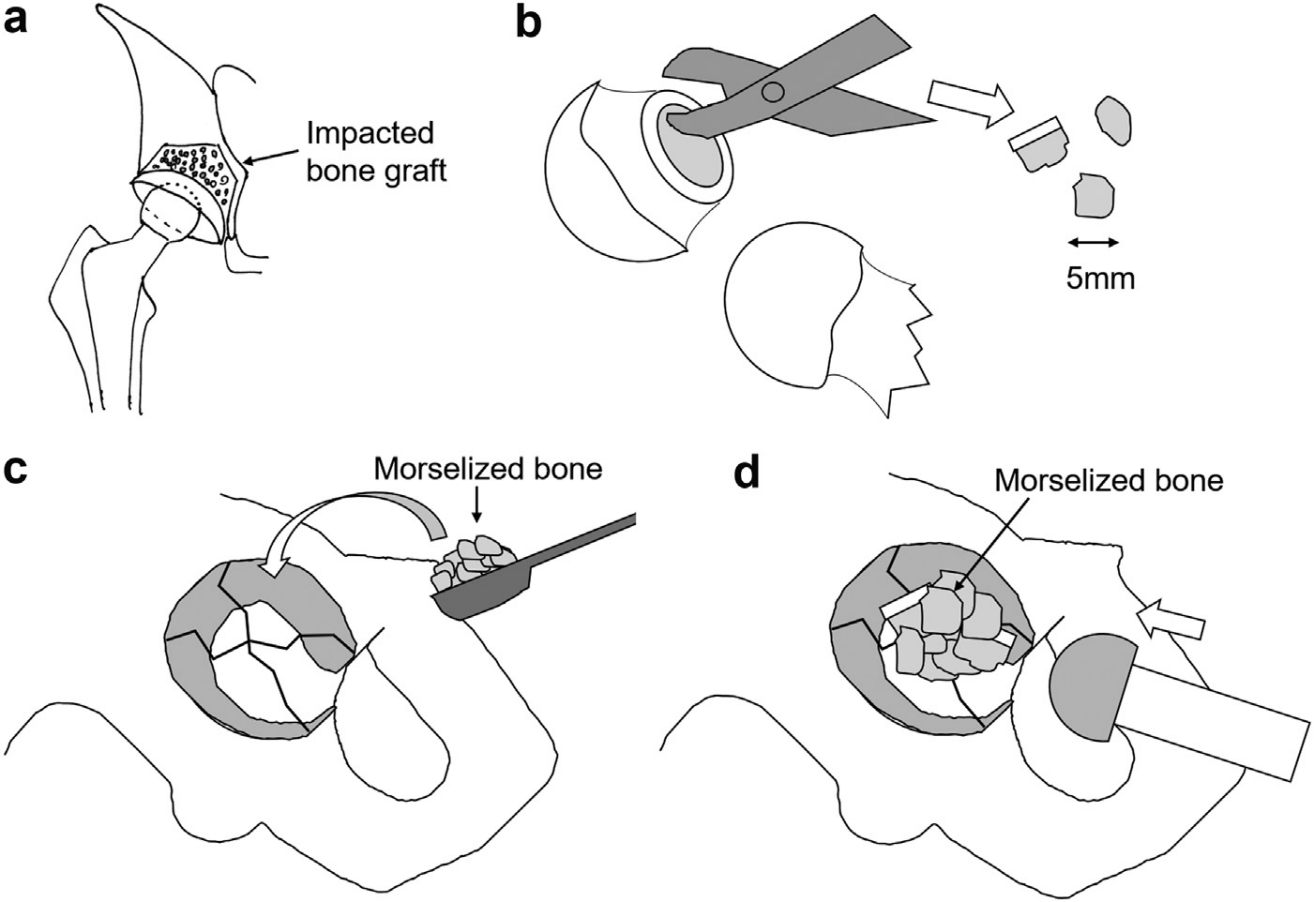
Illustration of an impacted bone graft of the acetabulum. (a) This was the preoperative plan of a THA with an impacted bone graft. (b) The 5- to 10-mm bone graft was morselized from the resected femoral head and/or allograft stored at −80°C for at least 3 months. (c) Some of the morselized bone grafts were put on the acetabular cavity. (d) The morselized bone grafts were compressed to the acetabular surface using the outer head trial, and these procedures were repeated several times to form a spherical surface.

In all 3 cases, an X3 ultra-high-molecular-weight polyethylene acetabular cup (Stryker Orthopedics, Mahwah, NJ) was cemented into the newly formed acetabular cavity at approximately 45° of abduction and 20° of anteversion. The internal diameter of the acetabular component was 28 mm. After reaming and broaching the femoral canal, an Exeter V40 femoral stem (Stryker Orthopedics, Mahwah, NJ) was cemented at approximately 20° of anteversion. We used Simplex P bone cement with tobramycin (Stryker Limerick, Limerick, Ireland) for both the acetabulum and femur (Fig. 1).

We removed the postoperative drain tube the day after surgery. Range of motion training of the hip on the bed and toe-touch exercises were started the day after surgery for all the patients. In line with past reports, weight-bearing walking within the tolerance of pain was allowed from 4 weeks after the surgery in all cases [8]. All the patients underwent venous ultrasonography of the lower extremities 2 weeks after the surgery.

The clinical data of all the patients are summarized in Table 1, and the details of each case are described in the sections that follow. Hip function was evaluated using the Japanese Orthopaedic Association (JOA) hip score every 3 months until a year postoperatively and annually thereafter. The JOA hip score covers 4 categories and comprises a 100-point scale: pain (40 points), range of hip motion (20 points), ambulatory status (20 points), and activities of daily living (20 points) [9]. The mean operative time was 165 minutes (range, 141-186 minutes), and the mean intraoperative bleeding volume was 729 mL (range, 394-924 mL). The mean JOA hip score at the last follow-up visit was 63.3 (range, 33-81).

**Table 1:** Clinical data of patients.

Patient data	Patient 1	Patient 2	Patient 3	Mean
Age	83	79	85	82.3
Sex	Female	Female	Female	
BMI, kg/m^2^	20.8	19.4	18.0	19.4
Follow-up, mo	21	6	15	
ASA-PS	2	2	2	
Injury mechanism	Fall	Fall	Unknown	
Bleeding, mL	394	869	924	729
Operative time, min	141	168	186	165
Perioperative transfusion, IU	2	0	6	
Postoperative complications	-	Pneumonia	Hip dislocation	
JOA hip score, a week prior to the surgery	33	27	38	32.7
JOA hip score, at the last follow-up	76	33	81	63.3

ASA-PS, American Society of Anesthesia-Performance Status; BMI, body mass index; JOA, Japanese Orthopedic Association.

## Case 1

An 83-year-old woman with a body mass index (BMI) of 20.8 kg/m^2^ and a medical history of angina pectoris, uterine fibroids, and hypertension developed right hip pain after falling. She was diagnosed with a nondisplaced acetabular fracture at an orthopedic clinic. She was treated conservatively under a weight-bearing limit with a walker. However, her hip pain gradually worsened, and for her, it was difficult to ambulate about 6 weeks after the injury. She visited our hospital 2 months following the injury. We diagnosed a comminuted acetabular fracture with quadrilateral surface destruction and Paprosky 2C acetabular defects. Her American Society of Anesthesiologists score was II, and her JOA hip score was 33 on her first visit.

**Figure 2:**
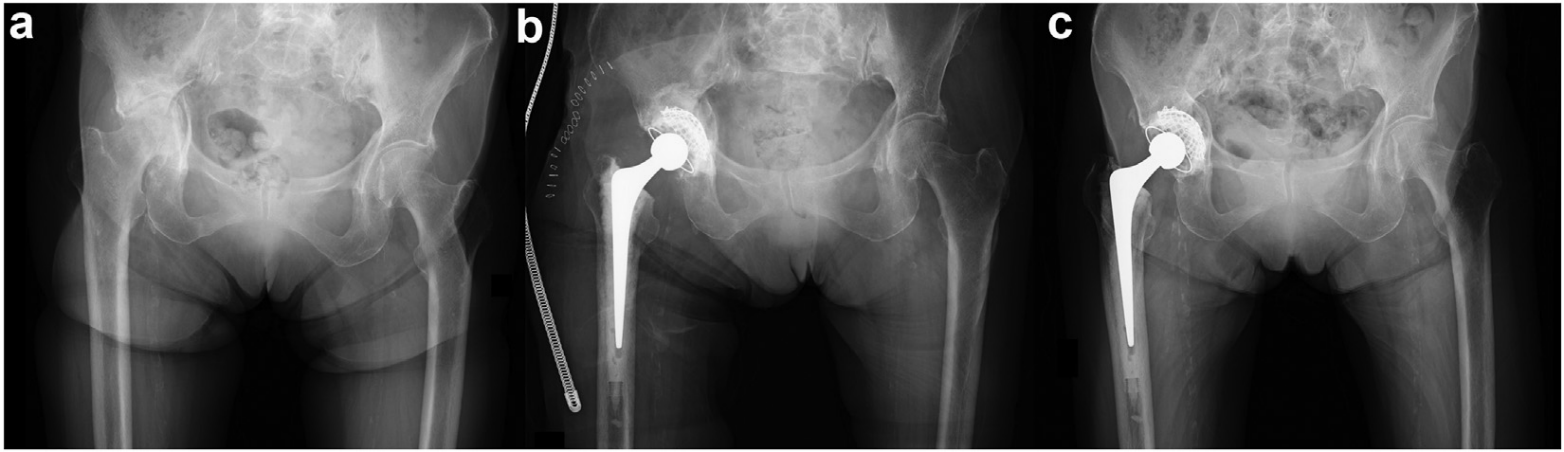
A case presentation (case 1). (a) Radiographic imaging at the patient’s first visit to our hospital showed a comminuted acetabular fracture with quadrilateral surface destruction as well as a femoral head fracture. Good callus formation on the acetabulum was evident. (b) Radiographic images after late THA with IBG 2 months after the initial trauma. (c) A radiographic image at the 3-year postoperative follow-up showing no component loosening or any subsidence of the acetabular cemented cup.

We performed THA with IBG 1 week after her first visit because the acetabular roof showed good callus formation on radiographic imaging. She was discharged to a rehabilitation center 6 weeks after the surgery and was using a walker to ambulate. Three months after the surgery, she was discharged from the rehabilitation center and was able to ambulate with a cane. At the 6-month follow-up, she was able to ambulate without walking aids, and her JOA hip score was 83. She experienced no significant postoperative complications, and radiographic imaging showed no prosthesis component loosening nor any subsidence of the acetabular cemented cup 3 years after the surgery. Her JOA hip score at the last follow-up was 76 points (Fig. 2).

## Case 2

A 79-year-old woman with a BMI of 19.4 kg/m^2^ and a medical history of left femoral trochanteric fracture, breast cancer, and tuberculosis developed right hip pain after a fall and was diagnosed with a displaced acetabular fracture and a femoral neck fracture at an orthopedic hospital. She was hospitalized and treated conservatively with minimal physiotherapy to the affected lower limb because her hip pain was mild. She continued to have difficulty ambulating and was largely wheelchair-bound. Her hip pain gradually got worse. She was referred to our hospital 6 weeks after the initial injury. Radiographic imaging of her hip showed a displaced acetabular fracture with quadrilateral surface destruction and Paprosky 2C acetabular defects as well as femoral neck and head fractures. Her American Society of Anesthesiologists score was II, and her JOA hip score was 27 points on her first visit.

**Figure 3:**
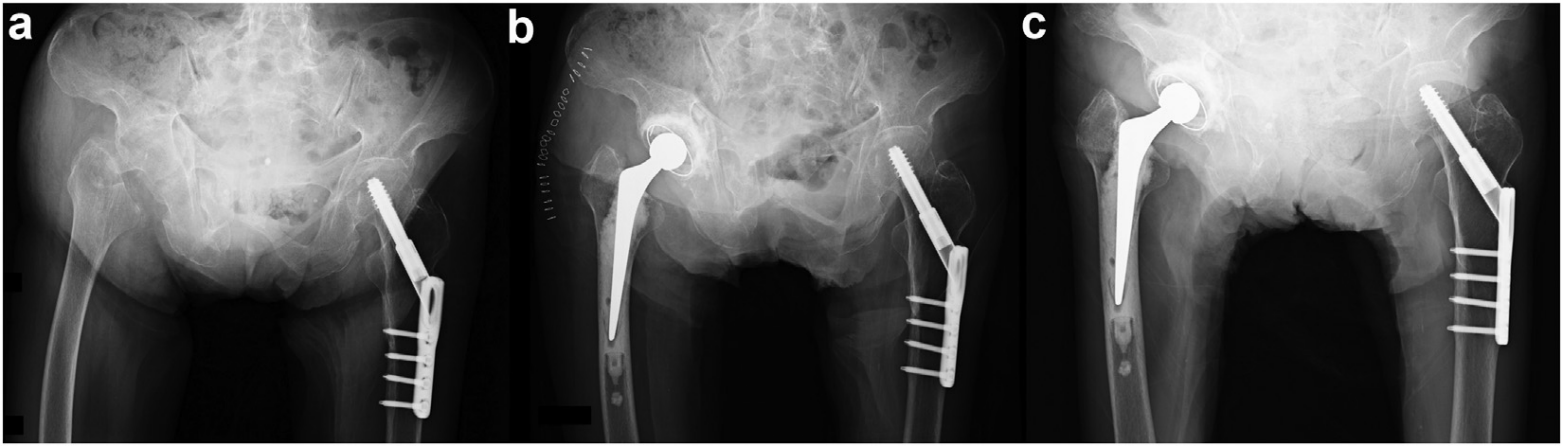
A case presentation (case 2). (a) Radiographic imaging at the patient’s first visit to our hospital showed a comminuted acetabular fracture with quadrilateral surface destruction as well as a femoral neck fracture. Good callus formation was evident on the acetabulum. (b) Radiographic images after late THA with IBG 2 months after the initial trauma. (d) Radiographic image at the 6-month postoperative follow-up showed no component loosening or any subsidence of the acetabular cemented cup. The patient died of pneumonia 2 weeks after our 6-month follow-up.

We performed THA with IBG due to good callus formation on the acetabular roof, which was confirmed on radiographic imaging performed 2 weeks after her first visit. She was discharged to a rehabilitation center 5 weeks after the surgery and was able to ambulate short distances with a walker. Three months after the surgery, she was discharged from the rehabilitation center and was able to ambulate with a walker. Her JOA hip score was 64 points. She was largely wheelchair-bound although her hip pain was well controlled with acetaminophen. She was subsequently hospitalized with pneumonia and died 2 weeks after her last follow-up (Fig. 3).

## Case 3

An 85-year-old woman with a BMI of 18.0 kg/m^2^ and a medical history of hypertension developed right hip pain without a history of trauma. She visited an orthopedic clinic and was diagnosed with no obvious fractures on radiographic imaging of the hip. Her hip pain gradually worsened, and she revisited the orthopedic clinic a week after her first visit. She was diagnosed with a displaced acetabular fracture and was referred to our hospital the following day. Radiographic imaging of the hip showed a displaced acetabular fracture with quadrilateral surface destruction and Paprosky 2C acetabular defects as well as a femoral head fracture. Her American Society of Anesthesiologists score was II. We expected it to be challenging to fix this type of acetabular fracture and stabilize it with a cage or ORIF, so we planned a late THA with IBG after confirming callus formation on the acetabulum. The patient was hospitalized and treated conservatively with traction for 2 weeks and discharged to a rehabilitation center thereafter. She had been using a non-weight-bearing wheelchair with minimal physiotherapy to the affected lower limb prior to the surgery.

**Figure 4:**
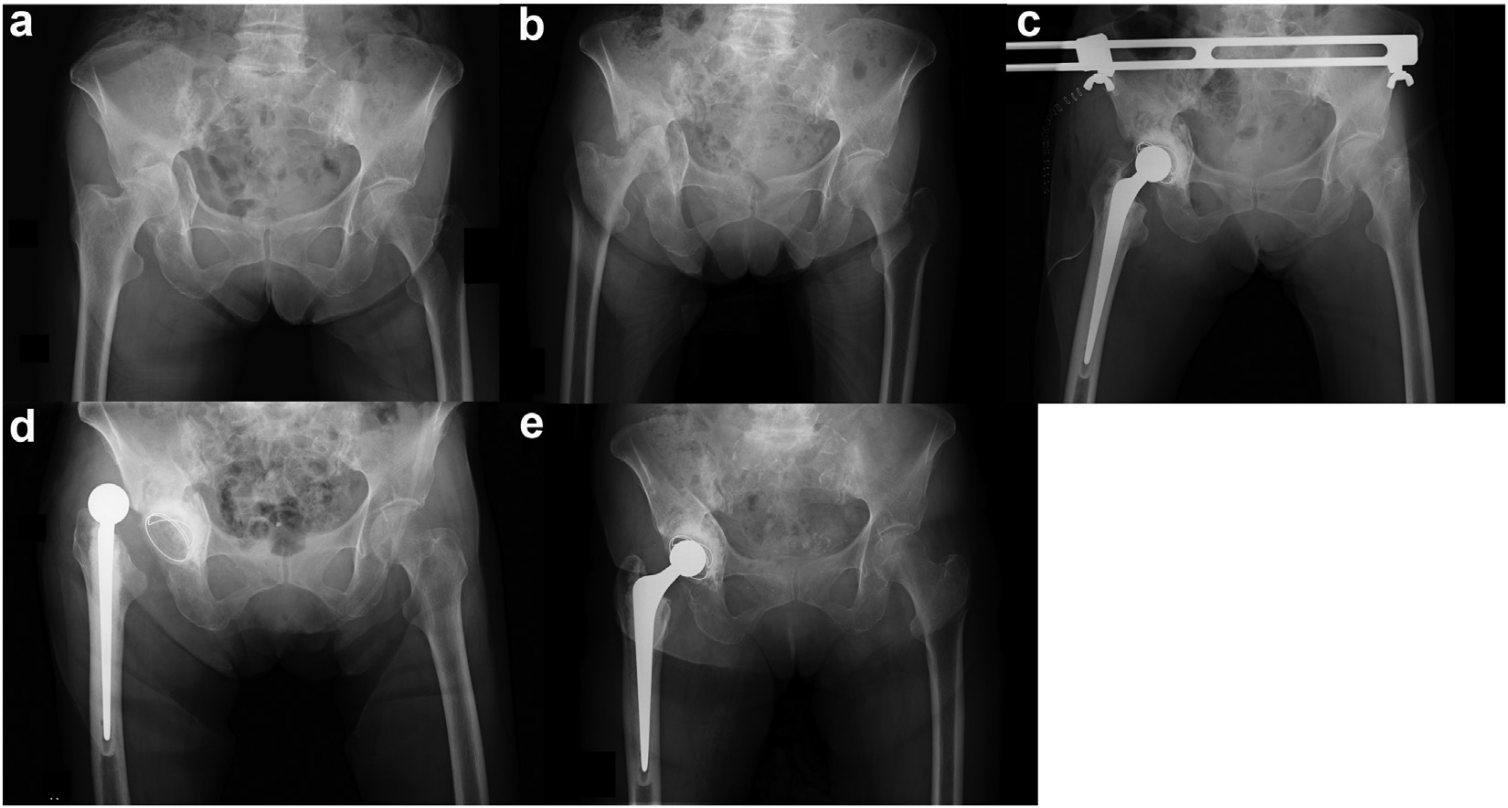
A case presentation (case 3). (a) The radiographic imaging at the patient’s first visit to our hospital showed a comminuted acetabular fracture with quadrilateral surface destruction as well as a femoral neck fracture. Good callus formation was evident on the acetabulum. (b) Radiographic imaging 2 months after the patient’s initial injury showed good callus formation. (c) Radiographic imaging after late THA with IBG 2 months after the initial trauma. (d) The patient experienced an anterior dislocation due to hyperextension at 2 months postoperatively. (e) Radiographic imaging at the 3-year follow-up showed no component loosening or any subsidence of the acetabular cemented cup.

She was transferred from the rehabilitation center to our hospital 8 weeks after her first admission to our hospital. Her JOA hip score at that time was 38 points. Radiographic imaging confirmed good callus formation on the acetabulum, and we performed THA with IBG. She was discharged to the rehabilitation center 6 weeks after the surgery and used a walker to ambulate. She experienced an anterior prosthetic hip dislocation when taking something from a high shelf. We performed a closed reduction for this hip dislocation. She was treated conservatively with a rigid functional brace that restricted overextension, and there was no subsequent recurrence. The patient was discharged from the rehabilitation center 4 months following the surgery and used a cane for ambulation. At the 6-month postoperative follow-up, she was able to ambulate without walking aids, and her JOA hip score was 80 points. Radiographic imaging 3 years after the surgery showed no prosthesis component loosening or any subsidence of the acetabular cemented cup. Her JOA hip score at the last follow-up was 81 points (Fig. 4).

## Discussion

ORIF for acetabular fractures has shown satisfactory outcomes in young patients [2] but less favorable results in the elderly. The best treatment for acetabular fractures in the elderly remains controversial, and no consensus or guidelines exist. Kreder et al. performed a retrospective study of 128 patients who underwent ORIF for acetabular fractures and found that 54% of the patients older than 50 years with marginal impaction and wall comminution required a revision surgery with THA [10]. Clarke-Jenssen et al. reported a 0% hip survival rate at 3 years in patients older than 60 years with a femoral head injury and acetabular dome impaction [3].

Recently, instead of only ORIF, alternative methods have been considered. Borg et al. showed a higher short-term hip joint survival rate for acute THA combined with internal fixation for acetabular fractures in older patients than for ORIF alone (100% vs 28.6%, respectively) [4]. Enocson and Blomfeldt demonstrated that primary THA with a Burch-Schneider cage and autologous bone graft for comminuted acetabular fractures in the elderly provided good functional and radiographic results [5]. In their study, Boelch et al. found that primary THA for osteoporotic acetabular fractures was a more advantageous option than ORIF; however, their study sample size was small [6]. These reports indicate that primary THA may be a good alternative treatment option for fragility acetabular fractures. However, this procedure is so complicated that it requires long operative times (mean operative time, 188-228 minutes) and causes high intraoperative bleeding (mean blood loss, 700-1155 mL) [5,6]. When a patient presents in the subacute phase, there is a high possibility of increased bleeding due to the presence of adhesions to the tissues, and when the origin of the injury is unknown, it is likely to be difficult to fix and stabilize with ORIF. We prefer this method in cases only with acetabular comminuted fractures. In this study, the mean operative time was 165 minutes, and postoperative bleeding was 729 mL. Notwithstanding, this is for reference due to the small number of cases in this study.

IBGs of the acetabulum in total hip replacements were initially reported by Hastings and Parker in 1975 and are widely employed for restoring acetabular bone defects in hip replacements [11]. Bradford et al. treated Paprosky 3B acetabular defects with IBG combined with a reinforcement mesh and demonstrated the effectiveness of the procedure [7]. Re-revision of the THA with IBG for aseptic loosening (Paprosky 3A or 3B) resulted in good functional outcomes and a low postoperative failure rate of the acetabular component in a previous study [12]. Although these studies included younger patients, we believe that acetabular defects after aseptic component loosening or large bone defects such as those classified as Paprosky 3B are similar to osteoporotic acetabular defects. We consider this technique an effective method for reconstructing fragility comminuted acetabular fractures that are cumbersome to fix and stabilize and for reinforcing the fixation of an acetabular cup.

## Summary

In this report, we have presented 3 case studies of late THA with IBG for acetabular fractures. Two of the 3 patients achieved good functional and radiographic outcomes at the 3-year follow-up. Although this procedure requires patients who can tolerate the delay in fixation, it can be a promising treatment option in elderly patients who present in the subacute phase or who have fracture types that are expected to be difficult to fix and stabilize with a spanning cage or ORIF.

## Conflicts of interest

The authors declare there are no conflicts of interest.

For full disclosure statements refer to https://doi.org/10.1016/j.artd.2022.10.004.

## Informed patient consent

The authors confirm that informed consent has been obtained from the involved patients or, if appropriate, from the parent, guardian, or power of attorney of the involved patients and that they have given approval for this information to be published in this article.

## Appendix A Supplementary Data

Conflict of Interest Statement for Kuwahara

Conflict of Interest Statement for Yamauchi

Conflict of Interest Statement for Mitsuya

Conflict of Interest Statement for Takeda
